# Hydroxyurea-induced Tooth Discoloration

**DOI:** 10.4274/tjh.galenos.2019.2019.0275

**Published:** 2020-02-20

**Authors:** Muhammed Okuyucu, Memiş Hilmi Atay

**Affiliations:** 1 Ondokuz Mayıs University Faculty of Medicine, Department of Internal Medicine, Samsun, Turkey; 2 Ondokuz Mayıs University Faculty of Medicine, Department of Internal Medicine, Division of Hematology, Samsun, Turkey

**Keywords:** Hydroxyurea, Tooth discoloration, Myeloproliferative disorders

## To the Editor,

Hydroxyurea is an antineoplastic agent that inhibits DNA synthesis by inhibiting the enzyme ribonucleotide reductase that catalyzes the conversion of ribonucleotide diphosphate to deoxyribonucleotide triphosphate, which is used for DNA synthesis and repair. It is often used for the treatment of myeloproliferative disorders [1]. Most commonly, fever, pneumonitis, and dermatological (eczema, ulceration), gastrointestinal (mucositis), and hematological adverse effects are reported in patients receiving hydroxyurea. Oral lesions and brown staining of the fingers and toes has been reported rarely [2,3,4].

**Figure 1:**
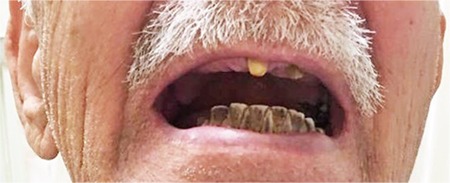
Hydroxyurea induced tooth discoloration in the patient.

An 82-year-old male patient was referred to the hematology clinic with an elevated platelet count. Laboratory investigations revealed a white blood cell count of 17,000/mm^3 ^(reference range: 3,900-10,900/mm^3^), hemoglobin of 17.5 g/dL (13.5-16.9 g/dL), hematocrit of 54% (40%-49%), and platelet count of 1,200,000/µL (166,000-308,000/µL). Upon physical examination, the spleen was palpable (3 cm below the costal margin). A peripheral smear showed a tear drop cell and giant platelets. Grade 3 reticulin fibrosis was observed upon bone marrow examination. With the diagnosis of myelofibrosis and hydroxyurea (1.5 g/day), therapy was initiated. Upon follow-up after 3 months of treatment, black discoloration of the teeth was noted in the patient (Figure 1). The black pigmentation was absent on his fingernails and toenails and he did not have a history of smoking, nor had he started a new medication. The patient was examined by a dentist, who thought that the discoloration might have been caused by the drug. Since the patient had a high risk of thrombosis (older age, rising white blood cell and platelet counts), hydroxyurea was not stopped. Because the tooth discoloration was not a life-threatening situation, the hydroxyurea dose was not modified. The patient was referred to the dentist for dental cleaning. We concluded that the black discoloration of the teeth associated with hydroxyurea was a rare and unprecedented clinical presentation.

Drug-induced tooth discoloration is categorized into two types: extrinsic and intrinsic. The extrinsic type involves staining of the outer surface of the tooth. Extrinsic factors include smoking, excessive consumption of tea and coffee, and medications. Solutions and antimicrobial preparations (amoxicillin-clavulanic acid, ciprofloxacin, linezolid) containing iron have been most prevalently associated with extrinsic discoloration of the teeth. In intrinsic discoloration, stains are deposited within the enamel of dentin during the development of tooth (e.g., tetracycline stains). In extrinsic discoloration, stains are removed by dental scaling and polishing [5]. It has been known that cutaneous toxicities generally develop after prolonged hydroxyurea usage. However, some publications in the literature reported that cutaneous toxicity can occur within 3 months [6]. In this case, we thought that with increasing age, erosion-related thinning of the outer layer of the tooth and poor oral hygiene could have caused discoloration of the tooth earlier.

In conclusion, this is the first case of tooth discoloration associated with chronic use of hydroxyurea to be reported in the literature. Clinicians should be aware of this uncommon adverse effect of hydroxyurea.
